# Association between the uric acid to high-density cholesterol ratio and abdominal aortic calcification: Evidence from NHANES 2013 to 2014

**DOI:** 10.1097/MD.0000000000047456

**Published:** 2026-01-30

**Authors:** Jia Guo, Ying Zhang, Yingchun Huang, Xing Li, Zhengqiang Tang, Xiaoqian Zeng, Zhi Fang

**Affiliations:** a Department of Cardiovascular Surgery, West China Hospital, Sichuan University, Chengdu, Sichuan, China; b West China School of Nursing, Sichuan University, Chengdu, Sichuan, China.

**Keywords:** abdominal aortic calcification, cross-sectional study, HDL cholesterol, NHANES database, UHR index, uric acid

## Abstract

Previous studies have suggested associations between uric acid (UA), high-density lipoprotein cholesterol (HDL-C), and vascular calcification. The UA to HDL-C ratio (UHR) has recently emerged as a novel marker for cardiovascular risk. This study aimed to investigate the relationship between UHR and abdominal aortic calcification (AAC). Data were obtained from the 2013 to 2014 National Health and Nutrition Examination Survey and included participants aged 40 years and older with available data on UA, HDL-C, and AAC. Participants were categorized into quartiles based on UHR levels. AAC was evaluated using the Kauppila scoring system, with severe AAC defined as a score > 6. Weighted multivariable linear and logistic regression analyses were performed to assess the association between UHR, AAC scores, and severe AAC. Restricted cubic spline models were used to explore potential nonlinear relationships. A total of 3009 participants were included. Higher UHR was positively associated with AAC scores after full adjustment (β = 3.43, 95% confidence interval [CI]: 0.45–6.41, *P* = .024). Higher UHR was positively associated with AAC scores in all models, including the fully adjusted model (model 4: β = 3.43, 95% CI: 0.45–6.41, *P* = .024). Participants in the highest UHR quartile (Q4) had AAC scores ~0.70 units higher than those in the lowest quartile (Q1). With respect to severe AAC, higher UHR was significantly associated with increased risk in unadjusted (model 1: odds ratio = 17.93, 95% CI: 2.04–157.42, *P* = .009) and partially adjusted (model 2: odds ratio = 24.74, 95% CI: 1.94–315.34, *P* = .013) models, but not after full adjustment (model 3: *P* = .185; model 4: *P* = .365). Restricted cubic spline analysis revealed a linear relationship between UHR and AAC scores (*P* for overall association = 0.02, *P* for nonlinearity = 0.741). Elevated UHR was independently associated with higher AAC scores but not with severe AAC adjustment for potential confounders. These findings suggest that UHR may serve as a useful marker for assessing the severity of AAC in adults aged 40 years and older.

## 1. Introduction

Cardiovascular disease (CVD) imposes a substantial burden on healthcare systems and economies and remains a leading cause of complications and mortality worldwide.^[1]^ Vascular calcification, defined as abnormal mineral deposition within blood vessels, is commonly observed in patients with chronic kidney disease and coronary artery disease (CAD).^[2,3]^ This process significantly increases the risk of cardiovascular events. Among vascular sites, the abdominal aorta is particularly prone to calcification, with the prevalence of abdominal aortic calcification (AAC) rising with age. AAC has been recognized as an important predictor of cardiovascular-related morbidity and mortality.

Uric acid (UA), the final product of purine metabolism, has been identified as an independent predictor of CVD, including myocardial infarction, stroke, heart failure, hypertension, and atrial fibrillation.^[4]^ A prospective cohort study demonstrated that elevated UA levels are associated with a higher risk of cardiovascular-related death, even after adjustment for age.^[5]^ In addition, a UA concentration of 5.6 mg/dL has been proposed as the threshold for hyperuricemia in patients with diabetes, predicting both all-cause and CVD-specific mortality.^[6]^ Conversely, epidemiological studies consistently show that higher levels of high-density lipoprotein cholesterol (HDL-C) are associated with reduced CVD risk. HDL-C not only promotes reverse cholesterol transport but also provides anti-inflammatory and antioxidant protection.^[7]^

The UA-to-HDL cholesterol ratio (UHR) has recently emerged as a novel biomarker of metabolic status and inflammation.^[8]^ Prior studies have linked UHR to hypertension, insulin resistance, and CVD-related mortality.^[9–12]^ UHR is thought to reflect the balance between proinflammatory and anti-inflammatory mechanisms. Higher UHR values have been associated with adverse outcomes, including arterial stiffness, renal dysfunction, atherosclerosis, and ischemic heart disease.^[13–15]^

Recently, 2 cross-sectional studies have explored the relationship between UHR and AAC using National Health and Nutrition Examination Survey (NHANES) 2013 to 2014 data. Li and Bai^[16]^ reported a positive association between UHR and AAC risk using logistic and linear regressions, stratified analyses, and receiver operating characteristic curves. Li et al^[17]^ also found a significant linear association between UHR and AAC scores, and a nonlinear dose–response relationship, with subgroup interaction by sex and diabetes status. Although these studies establish a potential relationship, several questions remain unanswered. For instance, they do not fully address whether the UHR-AAC association is robust after comprehensive adjustment for metabolic and cardiovascular covariates, whether the association holds across stratified quartiles of UHR, or whether a continuous dose–response relationship (via restricted cubic splines) persists after adjustment.

Therefore, the present study aimed to further investigate the association between UHR and AAC by incorporating quartile-based and continuous modeling, extensive multivariable adjustments, restricted cubic spline analyses, and stratified subgroup evaluations. Through a more comprehensive and robust analysis of this relationship, our study seeks to deepen current insights into the potential clinical role of UHR in vascular calcification.

## 2. Methods

### 2.1. Study design

Data were obtained from the NHANES, a cross-sectional survey conducted by the National Center for Health Statistics. All NHANES datasets are publicly available on the official website (https://www.cdc.gov/nchs/nhanes/).

**Figure 1.**
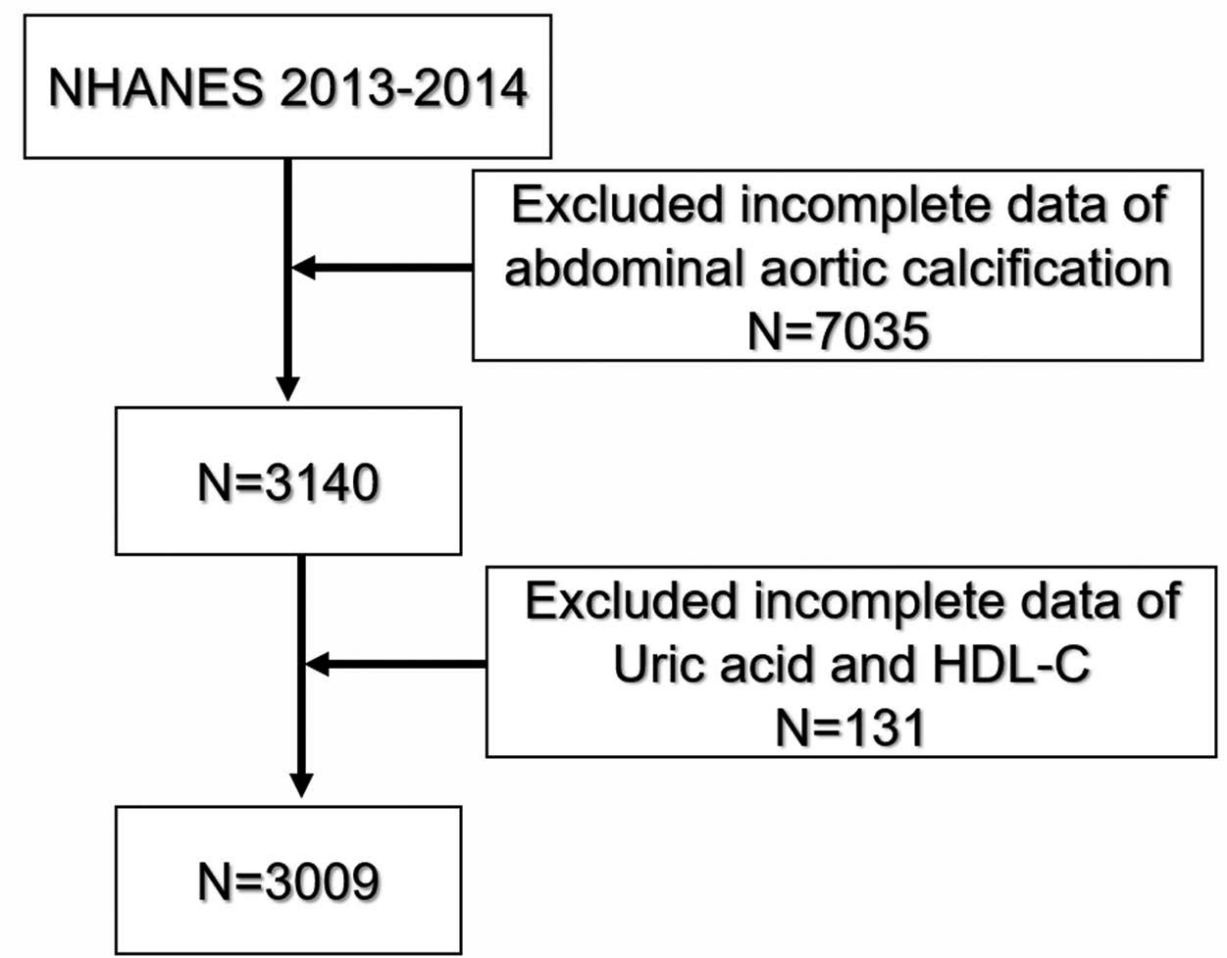
Flow chart of participant selection from NHANES 2013 to 2014. NHANES = National Health and Nutrition Examination Survey.

This study utilized data from the 2013 to 2014 NHANES survey cycle, which included detailed information on UA, HDL-C, and AAC scores. Participants were eligible for inclusion if they had complete data on UA, HDL-C, and AAC. Individuals with missing AAC data (including participants younger than 40 years, pregnancy, n = 7035) or UHR data (n = 131) were excluded. The final analytic sample comprised 3009 participants (Fig. 1). The NHANES protocol was approved by the National Center for Health Statistics Ethics Review Board, and written informed consent was obtained from all participants. Because the present study involved secondary analysis of publicly available, de-identified NHANES data, additional ethical approval was not required.

### 2.2. UA to HDL-C ratio

Morning blood samples were collected and analyzed to determine HDL-C and UA concentrations. HDL-C was measured using a direct enzymatic colorimetric method on a Beckman Coulter DxC800 analyzer (Beckman Coulter Inc., Brea) with manufacturer-supplied reagents and calibrators. A magnesium sulfate/dextran solution was added to the sample to form soluble complexes with non-HDL cholesterol, preventing interference with subsequent reagents. Polyethylene glycol esterase was then used to hydrolyze HDL cholesterol esters into free HDL cholesterol. The resulting hydrogen peroxide reacted with 4-aminoantipyrine and a hydrogen sulfide donor, producing a purple-blue dye. HDL-C concentration was quantified photometrically at 600 nm. Serum UA levels were determined on the same analyzer using a timed endpoint uricase method, in which UA is oxidized to allantoin and hydrogen peroxide. The hydrogen peroxide then reacted with 4-aminoantipyrine and 3,5-dichloro-2-hydroxybenzenesulfonate in a peroxidase-catalyzed reaction, generating a colored compound. UA concentration was determined photometrically at 520 nm. The UHR was calculated as the ratio of UA concentration (mg/dL) to HDL-C concentration (mg/dL).

### 2.3. Abdominal aortic calcification

This study examined 2 primary outcomes: the AAC score and the prevalence of severe AAC. The extent of AAC was assessed using lateral spine images obtained by dual-energy X-ray absorptiometry with a Hologic Discovery A densitometer (Hologic Inc., Bedford). Scans were performed by trained NHANES technicians following standardized acquisition, calibration, and quality control protocols.^[18]^ The severity of AAC was quantified using the Kauppila scoring method, which evaluates calcified deposits along the anterior and posterior aortic walls adjacent to the L1 to L4 vertebrae. Segment scores (0–6) were summed to yield a total AAC score ranging from 0 to 24 (NHANES variable DXXAAC24). Severe AAC was defined as a total score > 6, consistent with thresholds established in previous research.^[19]^

### 2.4. Covariates

To account for potential confounding, multivariable regression models incorporated a range of covariates selected based on prior literature. These included age, gender, race, body mass index (BMI), hypertension, diabetes, CAD, neutrophil count, lymphocyte count, total cholesterol, triglycerides, creatinine clearance (CCr), total bilirubin, calcium, aspartate aminotransferase (AST), alanine aminotransferase (ALT), lactate dehydrogenase, systolic blood pressure, and diastolic blood pressure. Categorical variables included gender, race, hypertension, diabetes, and CAD. Detailed measurement protocols for these covariates are available in the publicly accessible 2013 to 2014 NHANES dataset. Missing values were addressed using interpolation methods implemented in R software.

### 2.5. Statistical analysis

All statistical analyses were conducted in accordance with NHANES protocols. Continuous variables were reported as means with standard deviations, while categorical variables were expressed as percentages. Differences across UHR categories were evaluated using weighted Student *t* tests for continuous variables and weighted chi-square tests for categorical variables. Linear regression analyses were performed with AAC score as a continuous dependent variable to evaluate the association between UHR and the overall severity of calcification. Additionally, logistic regression analyses were conducted using severe AAC as a binary outcome to assess whether UHR was associated with the risk of severe calcification.

To examine the independent association between UHR and AAC outcomes, including both AAC scores and the prevalence of severe AAC, 4 multivariable regression models were constructed: model 1 was unadjusted; model 2 adjusted for gender, age, and race; model 3 further adjusted for BMI, hypertension, diabetes, CAD, systolic blood pressure, and diastolic blood pressure; and model 4 additionally adjusted for neutrophil count, lymphocyte count, cholesterol, triglycerides, CCr, total bilirubin, calcium, AST, ALT, and lactate dehydrogenase. Subgroup analyses were performed by race, gender, age, BMI, hypertension, diabetes, and CAD, with interaction terms tested to assess the heterogeneity of associations across subgroups. Restricted cubic spline models with 3 knots were used to flexibly examine and visualize the relationship between UHR and AAC. Statistical significance was defined as a 2-sided *P*-value < .05, and all analyses were conducted using R version 4.4.1.

## 3. Results

### 3.1. Baseline characteristics of participants

The baseline characteristics of participants according to UHR quartiles are summarized in Table 1. A total of 3009 individuals were included, of whom 48.16% were male and 51.84% were female. Participants were classified into 4 groups based on UHR: Q1 (<0.08), Q2 (0.08–0.10), Q3 (0.10–0.14), and Q4 (>0.14). Significant differences across UHR groups were observed in gender, BMI, hypertension, diabetes, CAD, systolic blood pressure, WBC, neutrophil count, lymphocyte count, total cholesterol, CCr, calcium, AST, ALT, AAC scores, and the prevalence of severe AAC. Both AAC scores and the frequency of severe AAC increased progressively with higher UHR quartiles. The overall mean AAC score was 1.64 ± 3.52, with mean values of 1.44 ± 3.52, 1.39 ± 3.10, 1.64 ± 3.42, and 2.08 ± 4.05 for Q1 through Q4, respectively. Severe AAC was present in 10.87% of participants, with higher rates observed in the Q3 and Q4 groups.

**Table 1:** Characteristics of participants.

Variables	Total (n = 3009)	Q1 (n = 750)	Q2 (n = 753)	Q3 (n = 752)	Q4 (n = 754)	*P*
Age	58.64 ± 12.01	58.23 ± 12.10	58.21 ± 11.88	58.90 ± 12.02	59.20 ± 12.03	.284
Gender, n (%)						<.001
Male	1449 (48.16)	138 (18.40)	303 (40.24)	430 (57.18)	578 (76.66)	
Female	1560 (51.84)	612 (81.60)	450 (59.76)	322 (42.82)	176 (23.34)	
Race, n (%)						.154
Mexican American	396 (13.16)	87 (11.60)	110 (14.61)	104 (13.83)	95 (12.60)	
Other Hispanic	284 (9.44)	66 (8.80)	85 (11.29)	64 (8.51)	69 (9.15)	
Non-Hispanic White	1338 (44.47)	358 (47.73)	302 (40.11)	341 (45.35)	337 (44.69)	
Non-Hispanic Black	575 (19.11)	139 (18.53)	137 (18.19)	152 (20.21)	147 (19.50)	
Other race	416 (13.83)	100 (13.33)	119 (15.80)	91 (12.10)	106 (14.06)	
BMI	28.46 ± 5.56	25.76 ± 5.14	28.29 ± 5.55	29.31 ± 5.27	30.46 ± 5.18	<.001
Hypertension, n (%)						<.001
Yes	1426 (47.44)	279 (37.20)	329 (43.87)	393 (52.26)	425 (56.37)	
No	1580 (52.56)	471 (62.80)	421 (56.13)	359 (47.74)	329 (43.63)	
Diabetes, n (%)						<.001
Yes	494 (17.09)	67 (9.14)	112 (15.51)	144 (19.89)	171 (24.05)	
No	2396 (82.91)	666 (90.86)	610 (84.49)	580 (80.11)	540 (75.95)	
CAD, n (%)						<.001
Yes	159 (5.30)	23 (3.07)	36 (4.79)	31 (4.13)	69 (9.21)	
No	2842 (94.70)	726 (96.93)	716 (95.21)	720 (95.87)	680 (90.79)	
SBP	126.56 ± 18.22	124.77 ± 19.32	125.95 ± 17.87	126.96 ± 17.00	128.55 ± 18.47	.001
DBP	70.13 ± 13.16	69.27 ± 12.29	69.68 ± 11.84	70.86 ± 13.66	70.70 ± 14.64	.074
WBC	7.14 ± 2.18	6.56 ± 2.00	7.01 ± 2.09	7.35 ± 2.27	7.63 ± 2.21	<.001
Neutrophil	4.22 ± 1.74	3.86 ± 1.61	4.14 ± 1.72	4.37 ± 1.82	4.49 ± 1.74	<.001
Lymphocyte	2.09 ± 0.74	1.97 ± 0.76	2.04 ± 0.68	2.13 ± 0.73	2.21 ± 0.78	<.001
Total cholesterol	195.81 ± 42.73	203.02 ± 38.05	195.67 ± 41.60	194.46 ± 44.66	190.14 ± 45.29	<.001
TC	5.03 ± 1.07	5.20 ± 0.96	5.02 ± 1.04	5.02 ± 1.13	4.89 ± 1.13	<.001
LDH	128.43 ± 25.87	129.34 ± 26.49	129.11 ± 24.74	127.46 ± 25.14	127.83 ± 27.05	.403
CCr	0.94 ± 0.52	0.81 ± 0.37	0.90 ± 0.66	0.95 ± 0.31	1.12 ± 0.62	<.001
TB	0.64 ± 0.31	0.62 ± 0.27	0.64 ± 0.39	0.66 ± 0.29	0.64 ± 0.26	.245
Ca	9.45 ± 0.37	9.47 ± 0.37	9.42 ± 0.35	9.49 ± 0.38	9.43 ± 0.37	<.001
AST	25.48 ± 13.99	24.28 ± 10.07	25.41 ± 18.16	25.10 ± 10.14	27.12 ± 15.63	<.001
ALT	24.63 ± 18.30	21.11 ± 12.85	24.54 ± 26.12	24.67 ± 13.09	28.17 ± 17.20	<.001
Insulin	13.13 ± 23.39	7.88 ± 6.94	11.23 ± 13.34	14.51 ± 17.93	19.47 ± 41.04	<.001
APoB	93.22 ± 25.20	84.70 ± 20.55	92.55 ± 24.71	95.64 ± 26.55	100.76 ± 26.10	<.001
AAC score	1.64 ± 3.52	1.44 ± 3.40	1.39 ± 3.10	1.64 ± 3.42	2.08 ± 4.05	<.001
Severe AAC						.024
Yes	327 (10.87)	71 (9.47)	69 (9.16)	85 (11.30)	102 (13.53)	
No	2682 (89.13)	679 (90.53)	684 (90.84)	667 (88.70)	652 (86.47)	

ALT = alanine aminotransferase, AST = aspartate aminotransferase, BMI = body mass index, CCr = creatinine clearance, DBP = diastolic blood pressure, DM = diabetes mellitus, LDH = lactate dehydrogenase, SBP = systolic blood pressure, TB = total bilirubin, TC = total cholesterol, TG = triglyceride, WBC = white blood cell.

### 3.2. Association between UHR and AAC

As shown in Table 2, UHR was positively associated with AAC scores. This association was significant in unadjusted (model 1: β = 4.22, 95% confidence interval [CI]: 1.70–6.75, *P* = .001), partially adjusted (model 2: β = 3.92, 95% CI: 1.40–6.45, *P* = .002), and further adjusted (model 3: β = 3.97, 95% CI: 1.09–6.85, *P* = .007) models. Importantly, the relationship remained significant in the fully adjusted model (model 4: β = 3.43, 95% CI: 0.45–6.41, *P* = .024), indicating that each unit increase in UHR corresponded to a 3.43-point increase in AAC scores. Participants in the highest UHR quartile (Q4) had AAC scores that were, on average, 0.70 units higher than those in the lowest quartile (Q1).

**Table 2:** Associations between UHR with AAC with linear regression.

Variables	Model1		Model2		Model3		Model4	
	β (95% CI)	*P*	β (95% CI)	*P*	β (95% CI)	*P*	β (95% CI)	*P*
UHR	4.22 (1.70 to 6.75)	.001	3.92 (1.40 to 6.45)	.002	3.97 (1.09 to 6.85)	.007	3.43 (0.45 to 6.41)	.024
UHR quantile								
Q1	0.00 (Reference)		0.00 (Reference)		0.00 (Reference)		0.00 (Reference)	
Q2	−0.05 (−0.41 to 0.30)	.767	0.03 (−0.30 to 0.36)	.841	0.05 (−0.30 to 0.40)	.794	0.05 (−0.30 to 0.41)	.765
Q3	0.20 (−0.16 to 0.55)	.280	0.22 (−0.12 to 0.55)	.212	0.19 (−0.18 to 0.56)	.315	0.21 (−0.17 to 0.59)	.281
Q4	0.64 (0.28 to 0.99)	<.001	0.66 (0.31 to 1.02)	<.001	0.72 (0.32 to 1.13)	<.001	0.70 (0.28 to 1.11)	.001

Model 1: Crude.

Model 2: Adjust: gender, age, race.

Model 3: Adjust: gender, age, race, BMI, hypertension, diabetes, CAD, SBP, DBP.

Model 4: Adjust: gender, age, race, BMI, hypertension, diabetes, CAD, neutrophil count, lymphocyte count, total cholesterol, TG, CCr, TB, Ca, AST, ALT, LDH, SBP, DBP.

ALT = alanine aminotransferase, AST = aspartate aminotransferase, BMI = body mass index, CAD = coronary artery disease, CCr = creatinine clearance, CI = confidence interval, DBP = diastolic blood pressure, LDH = lactate dehydrogenase, SBP = systolic blood pressure, TB = total bilirubin, TG = triglyceride, UHR = UA to HDL-C ratio..

With respect to severe AAC (Table 3), higher UHR was significantly associated with increased risk in unadjusted (model 1: odds ratio (OR) = 17.93, 95% CI: 2.04–157.42, *P* = .009) and partially adjusted models (model 2: OR = 24.74, 95% CI: 1.94–315.34, *P* = .013). However, these associations were no longer statistically significant after full adjustment for clinical and biochemical covariates (model 3: *P* = .185; model 4: *P* = .365), suggesting that the observed relationship between UHR and severe AAC may be largely explained by confounding factors.

**Table 3:** Associations between UHR with severe AAC for logistic regression.

Variables	Model 1		Model 2		Model 3		Model 4	
	OR (95% CI)	*P*	OR (95% CI)	*P*	OR (95% CI)	*P*	OR (95% CI)	*P*
UHR	17.93 (2.04–157.42)	.009	24.74 (1.94–315.34)	.013	8.34 (0.36–192.24)	.185	4.48 (0.17–115.47)	.365
UHR quantile								
Q1	1.00 (Reference)		1.00 (Reference)		1.00 (Reference)		1.00 (Reference)	
Q2	0.96 (0.68–1.37)	.840	1.06 (0.72–1.55)	.769	0.95 (0.62–1.46)	.818	0.95 (0.61–1.48)	.820
Q3	1.22 (0.87–1.70)	.244	1.29 (0.89–1.88)	.178	1.07 (0.69–1.67)	.767	1.11 (0.71–1.75)	.647
Q4	1.50 (1.08–2.06)	.014	1.65 (1.13–2.42)	.010	1.47 (0.92–2.35)	.103	1.46 (0.90–2.35)	.125

Model 1: Crude.

Model 2: Adjust: gender, age, race.

Model 3: Adjust: gender, age, race, BMI, hypertension, diabetes, CAD, SBP, DBP.

Model 4: Adjust: gender, age, race, BMI, hypertension, diabetes, CAD, neutrophil count, lymphocyte count, total cholesterol, TG, CCr, TB, Ca, AST, ALT, LDH, SBP, DBP.

ALT = alanine aminotransferase, AST = aspartate aminotransferase, BMI = body mass index, CAD = coronary artery disease, CCr = creatinine clearance, CI = confidence interval, DBP = diastolic blood pressure, LDH = lactate dehydrogenase, OR = odds ratio, SBP = systolic blood pressure, TB = total bilirubin, TG = triglyceride, UHR = UA to HDL-C ratio.

### 3.3. Subgroup analyses

**Figure 2.**
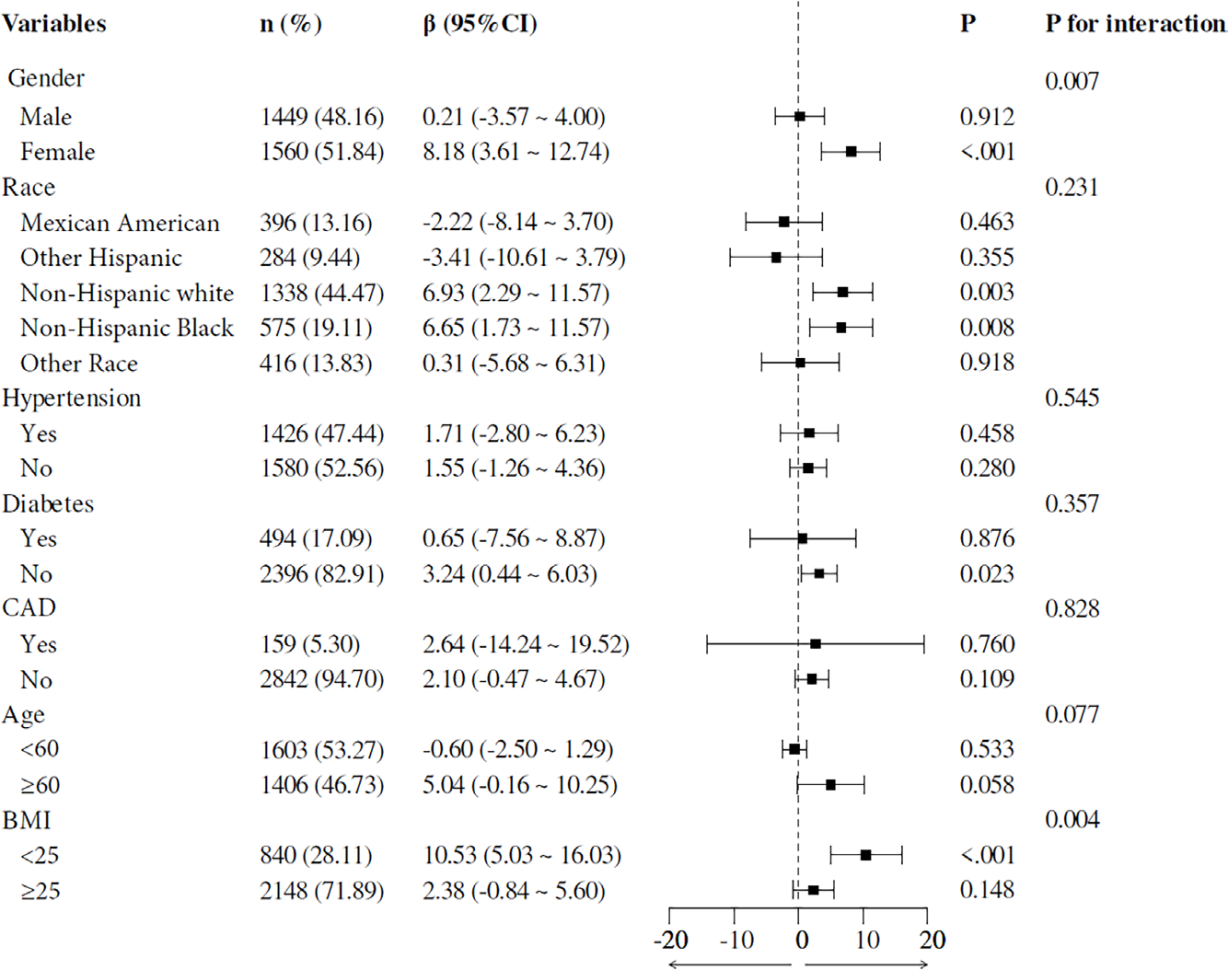
Subgroup analysis of the association between UHR and AAC. The analyses revealed a consistent association between UHR and AAC scores. Gender and BMI emerged as a notable significant interaction *P*-values affecting the association between UHR and AAC. AAC = abdominal aortic calcification, BMI = body mass index, UHR = uric acid-to-HDL cholesterol ratio.

Subgroup analyses were performed to evaluate whether the association between UHR and AAC varied by potential effect modifiers, including age, race, gender, BMI, hypertension, CAD, and diabetes (Table 4 and Fig. 2). Overall, the positive association between UHR and AAC scores was consistent across most subgroups, with no significant interactions observed for the majority of factors. However, sex showed a significant interaction effect (*P* for interaction = .007). The association was notably stronger in females (β = 8.18, 95% CI: 3.61–12.74, *P* < .001) compared with males (β = 0.21, 95% CI: −3.57–4.00, *P* = .912). BMI also demonstrated a significant interaction with the UHR-AAC association (*P* for interaction = .004).

**Table 4:** Subgroup analysis of the association between UHR and AAC scores.

Variables	n (%)	β (95% CI)	*P*	*P* for interaction
Gender				.007
Male	1449 (48.16)	0.21 (−3.57 to 4.00)	.912	
Female	1560 (51.84)	8.18 (3.61 to 12.74)	<.001	
Race				.231
Mexican American	396 (13.16)	−2.22 (−8.14 to 3.70)	.463	
Other Hispanic	284 (9.44)	−3.41 (−10.61 to 3.79)	.355	
Non-Hispanic White	1338 (44.47)	6.93 (2.29 to 11.57)	.003	
Non-Hispanic Black	575 (19.11)	6.65 (1.73 to 11.57)	.008	
Other race	416 (13.83)	0.31 (−5.68 to 6.31)	.918	
Hypertension				.545
Yes	1426 (47.44)	1.71 (−2.80 to 6.23)	.458	
No	1580 (52.56)	1.55 (−1.26 to 4.36)	.280	
DM				.357
Yes	494 (17.09)	0.65 (−7.56 to 8.87)	.876	
No	2396 (82.91)	3.24 (0.44 to 6.03)	.023	
CAD				.828
Yes	159 (5.30)	2.64 (−14.24 to 19.52)	.760	
No	2842 (94.70)	2.10 (−0.47 to 4.67)	.109	
Age				.077
<60	1603 (53.27)	−0.60 (−2.50 to 1.29)	.533	
≥60	1406 (46.73)	5.04 (−0.16 to 10.25)	.058	
BMI				.004
<25	840 (28.11)	10.53 (5.03 to 16.03)	<.001	
≥25	2148 (71.89)	2.38 (−0.84 to 5.60)	.148	

AAC = abdominal aortic calcification, BMI = body mass index, CAD = coronary artery disease, DM = diabetes mellitus, UHR = uric acid-to-HDL cholesterol ratio.

### 3.4. Restricted cubic spline model

**Figure 3.**
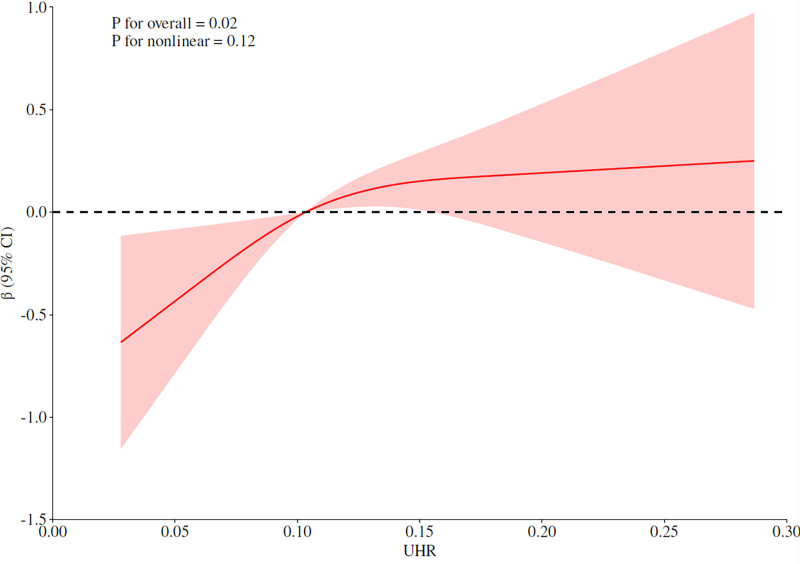
Association between UHR and AAC scores modeled by restricted cubic splines. The analysis showed a nearly linear relationship between the AIP index and the AAC score after adjustment for multiple potential covariates (*P* for overall = .02, *P* for nonlinear = .741). AAC = abdominal aortic calcification, AIP = atherogenic index of plasma, UHR = uric acid-to-HDL cholesterol ratio.

As illustrated in Figure 3, the restricted cubic spline model indicated a nearly linear relationship between UHR and AAC scores after adjustment for multiple covariates (*P* for overall association = .02, *P* for nonlinearity = .741).

## 4. Discussion

In this cross-sectional analysis of 3009 adults, we observed a significant association between elevated UHR and increased AAC scores, but no corresponding increase in the risk of severe AAC. These findings suggest that lowering UHR may help reduce the severity of AAC, if it does not directly affect the progression to severe calcification. Subgroup analyses indicated that this association was modified by gender and BMI, whereas no significant interactions were observed for race, age, hypertension, diabetes, or CAD. These findings suggest that the relationship between UHR and AAC may vary according to metabolic and physiological differences related to body composition and sex.

Elevated UA levels have long been linked to subclinical vascular injury, partly through their effects on vascular thickness, function, and structural integrity.^[20]^ Inflammation appears to play a central role in the vascular impact of UA. Monosodium urate crystals can activate the NALP3 inflammasome, triggering an inflammatory cascade via toll-like receptor signaling.^[21]^ UA also promotes neutrophil activation, leading to the release of proinflammatory mediators that exacerbate vascular inflammation.^[22]^ As the final metabolite of purine catabolism, UA has been shown to induce vascular smooth muscle cell migration and cytoskeletal remodeling, processes that contribute to arterial calcification.^[23]^ Chronic hyperuricemia further facilitates calcium deposition in the aortic media, promoting vascular stiffening and calcification over time.

An increase in UA is strongly associated with major risk factors for atherosclerosis, including hypertension, diabetes, metabolic syndrome, hypertriglyceridemia, vascular stiffness, and renal dysfunction.^[24–26]^ Li and Chen reported that individuals in the highest quartile of UA had nearly a 2-fold higher risk of subclinical atherosclerosis compared with those in the lowest quartile (OR = 1.956, 95% CI: 1.225–3.124).^[27]^ Threshold UA levels predictive of subclinical atherosclerosis were identified as 6.35 mg/dL for men and 5.25 mg/dL for women. Furthermore, a prior meta-analysis found that individuals with UA levels exceeding 357 µmol/L had a 1.8-fold greater risk of coronary artery calcification compared with those with normal UA levels.^[27]^

Lipid abnormalities also play a critical role in vascular calcification. Oxidized lipids can induce mineralization of vascular cells, while neutral lipids tend to accumulate within the endothelial layer.^[28]^ Over time, metabolic byproducts stimulate these lipids to undergo nonenzymatic modifications, leading to the formation of oxidized phospholipids, which promote calcification through multiple pathways in vitro.^[29]^ Serum lipidomic profiling has further identified fatty acids associated with cardiovascular calcification.^[30]^ For example, individuals with elevated coronary calcium scores show an increased abundance of fatty acyl chain lipids and reduced levels of 18-carbon fatty acyl chain phosphatidylcholines in serum. HDL particles appear to exert a protective role against vascular calcification. One study demonstrated an inverse association between HDL particles and coronary artery calcification in women with elevated estradiol (*P* for interaction = .007).^[31]^ Another study found that higher concentrations of large HDL particles were linked to a reduced risk of coronary artery calcification, although this effect was not independent of HDL-C levels.^[32]^

## 5. Study strengths and limitations

This study has several strengths, most notably the use of a comprehensive, standardized, nationwide dataset from NHANES. In addition, we sought to minimize confounding bias by adjusting for a wide range of covariates identified in prior research. Nonetheless, several limitations should be acknowledged. First, although multiple covariates were controlled for, residual confounding from unmeasured factors cannot be ruled out. Second, due to its cross-sectional design, this study cannot establish a causal relationship between UHR and AAC, highlighting the need for longitudinal research with larger sample sizes. Third, AAC measurements in NHANES are available only for the 2013 to 2014 cycle and for participants aged 40 years and older, which restricted both the sample size and the study period. Therefore, our findings may not be fully generalizable to younger populations or to more recent cohorts.

### 6. Conclusion

Elevated UHR was significantly associated with higher AAC scores but not with severe AAC. Given the adverse effects of UA and HDL-C on cardiovascular health, incorporating UHR into clinical evaluation may provide additional value in the assessment of patients with AAC. However, large-scale prospective studies are needed to confirm the causal relationship between UHR and AAC.

## Acknowledgments

The authors would like to thank the National Center for Health Statistics (NCHS) for providing access to the NHANES database and for their rigorous data collection protocols, which made this research possible. We are also grateful to the participants of the NHANES 2013–2014 cycle whose contributions underpin these findings. Special thanks to our institutional colleagues for their valuable input during the study design and manuscript preparation stages.

## Author contributions

**Data curation:** Jia Guo, Ying Zhang, Yingchun Huang, Xing Li, Zhengqiang Tang, Xiaoqian Zeng.

**Formal analysis:** Jia Guo.

**Investigation:** Jia Guo, Zhi Fang.

**Methodology:** Jia Guo, Zhi Fang.

**Supervision:** Zhi Fang.

**Validation:** Zhi Fang.

**Visualization:** Jia Guo, Ying Zhang, Yingchun Huang, Xing Li, Zhengqiang Tang, Xiaoqian Zeng.

**Writing – original draft:** Jia Guo.

**Writing – review & editing:** Zhi Fang.
